# Pathogen-derived biomarkers for active tuberculosis diagnosis

**DOI:** 10.3389/fmicb.2014.00549

**Published:** 2014-10-20

**Authors:** Paula Tucci, Gualberto González-Sapienza, Monica Marin

**Affiliations:** ^1^Sección Bioquímica, Facultad de Ciencias, Universidad de la RepúblicaMontevideo, Uruguay; ^2^Cátedra de Inmunología, DEPBIO, Instituto de Higiene, Facultad de Química, Universidad de la RepúblicaMontevideo, Uruguay

**Keywords:** *M. tuberculosis*, active infection, diagnosis, point of care, biomarkers

## Abstract

Tuberculosis (TB) is an infectious disease caused by members of *Mycobacterium tuberculosis* complex. Despite the availability of effective treatments, TB remains a major public health concern in most low and middle-income countries, representing worldwide the second leading cause of death from an infectious disease. Inadequate case detection and failures to classify the disease status hamper proper TB control. The limitations of the conventional diagnostic methods have encouraged much research activities in this field, but there is still an urgent need for an accurate point of care test for active TB diagnosis. A rapid, precise, and inexpensive TB diagnostic test would allow an earlier implementation of an appropriate treatment and the reduction of disease transmission. Pathogen-derived molecules present in clinical specimens of affected patients are being validated for that purpose. This short review aims to summarize the available data regarding biomarkers derived from *M. tuberculosis*, and their current usage in active TB diagnosis.

## INTRODUCTION

Tuberculosis (TB) is a major global health problem and one of the most important causes of death from an infectious disease. In 2012, 8.6 million people developed TB and 1.3 million died from the disease (WHO, 2013). Despite substantial investments and progress made in the implementation of Stop TB strategy by the World Health Organization (WHO), inadequate case detection and failures to accurately classify the disease status still hamper the control of TB (Wallis et al., 2010a; McNerney et al., 2012). Most humans infected with *Mycobacterium tuberculosis* (MTB) remain asymptomatic, and only a small proportion develops active TB disease. Typically the bacterium establishes a latent infection and the lifetime risk of developing the disease is near 10% unless an individual becomes immunocompromised, at which time the risk increases significantly (Young et al., 2008). TB is usually a chronic, slowly progressing disease that often keeps undiagnosed in patients for many years. In adults the most common form is chronic pulmonary TB, while extrapulmonary TB is especially common in children and HIV-coinfected patients (Jain, 2011).

The diagnosis of active TB is critical for controlling the disease. Conventional diagnostic methods of active TB include sputum smear microscopy (pulmonary TB) and *M. tuberculosis* isolation in bacteriological culture (currently the *gold standard* for definitive diagnosis of pulmonary and extrapulmonary TB). Although these methods are widely used for diagnosing TB, they suffer of specificity and sensitivity limitations (Tiwari et al., 2007; WHO, 2013), and microbiological culture takes several weeks to confirm a clinical diagnosis. Besides, both methods require highly skilled personnel and specialized laboratory infrastructure.

Recently, PCR based diagnostic methods were launched. The GeneXpert MTB/RIF (Cepheid Inc., USA) is a cartridge-based, automatic nucleic acid amplification test, for TB case detection and rifampicin resistance testing. It purifies, amplifies, and identifies targeted nucleic acid sequences in the TB genome, and provides results from unprocessed sputum samples in less than 2 h (Boehme et al., 2010). This assay showed a high sensitivity in both pulmonary (Boehme et al., 2010) and extrapulmonary TB (Hillemann et al., 2011). It was endorsed by WHO (2011a) for use in TB endemic countries. However, its high cost is a main barrier for the popularization of this new technology out of reference laboratories, in areas where the prevalence of the disease is higher (Steingart et al., 2012).

Unlike many other infectious diseases, in TB the specific antibody response is not so well understood. That is a consequence of the complex and highly evolved relationship of the pathogen with the immune system, its intracellular localization, and our partial understanding of its biology and host–pathogen interaction. This fact has largely frustrated the attempts to exploit the host response in antibody detection diagnostic assays, which could constitute an economical diagnostic alternative in low income countries (Young et al., 2008). A systematic review of the performance of various commercial serological tests has evidenced that this approach does not allow a reliable diagnosis of TB, reporting inconsistent, imprecise, and highly variable values for sensitivity and specificity (Steingart et al., 2011). Considering that situation in 2011, WHO issued a policy recommending not using these tests for the diagnosis of pulmonary and extra-pulmonary TB (WHO, 2011b).

In this context, a major focus of the WHO’s global plan to stop TB is the development of a simple and cost-effective diagnostic method to improve case detection (Pollock et al., 2013). Until the moment TB lacks an accurate rapid point-of-care (POC) diagnostic test that could distinguish individuals with active TB from those with latent disease or not infected (McNerney et al., 2012; Pollock et al., 2013). The failure of diagnostic tests based on the antibody response has greatly stimulated the interest in the development of rapid antigen detection methods (WHO, 2009). For that purpose much work is being performed aiming to discover and validate robust host and pathogen biomarkers of *M. tuberculosis* infection and disease (Doherty et al., 2009). This minireview covers *M. tuberculosis*-derived molecules identified in clinical samples of infected patients, and thus, could be proposed as diagnostic markers candidates. Bearing a potential correlation with the actual load of bacteria, they can be used both for diagnosis and treatment monitoring (WHO, 2009). Among these molecules, this review focuses on antigenic compounds that can be detected with antibodies in antigen detection assays, being particularly attractive for the development of POC diagnostic test. Other pathogen-derived markers [DNA, RNA, and molecules with enzymatic activities (Xie et al., 2012)] are out of the scope of this review.

## PATHOGEN-DERIVED BIOMARKERS

A biomarker is defined as a parameter that can be objectively measured as an indicator of normal or pathogenic biological processes, or as an indicator of pharmacological responses to therapeutic interventions (Wallis et al., 2010a,b; McNerney et al., 2012). In routine clinical care, biomarkers allow stratification of individual patients, thus helping to develop targeted interventions that might not otherwise produce overall benefits (Wallis et al., 2010a).

In an infectious disease biomarkers can be either host or pathogen-derived (McNerney et al., 2012). Human immunodeficiency virus (HIV) infection offers a prime example of biomarker’s utility both for initial diagnosis and for the evaluation of disease state and progression. After HIV infection, viral RNA, and p24 antigen detection are used to establish an early diagnosis. Afterward, to evaluate HIV progression, viral load is measured by viral RNA quantification, and disease evolution is evaluated through CD4+ cell counts (Constantine and Zink, 2005). In that case understanding of the pathogen dynamics and kinetics of the host immune response during the disease allowed the development of accurate diagnostic tests.

A main challenge in TB is to identify and validate consistent markers which could be translated into a specific and sensitive diagnostic test. Unfortunately, knowledge in that field is still partial, and requires a better understanding of the disease and the host-pathogen interaction (Young et al., 2008). It is expected that the identification of specific molecular markers would help to the development of an *in vitro* diagnostic test for *M. tuberculosis* active infection, which should be rapid, inexpensive, sensitive, and appropriate to be used in peripheral laboratories with low level of infrastructure.

## PRINCIPAL *M. tuberculosis* DERIVED BIOMARKERS

To be considered as targets for antigen detection assays, pathogen-derived molecules must reach the sample matrixes (sputum, urine, plasma, etc.) in detectable levels (Bekmurzayeva et al., 2013). To be valuable as diagnostic biomarkers these antigens should be specifically and ubiquitously detected in clinical samples of infected patients.

An antigen detection assay for TB could be performed using a variety of clinical specimens such us sputum, blood, urine, saliva, cerebrospinal fluid (CSF), and pleural fluid. Antigens that are shed from *M. tuberculosis* in infected tissues can be present in the body fluids surrounding these tissues wherefrom they can reach the blood circulation and be eliminated in urine, a highly practical specimen for diagnostic tests. Urine is safer to handle and less variable than sputum, besides it is easier to collect from both adults and children. Additionally, urine based assays could facilitate TB diagnosis in HIV co-infected patients, who normally have a low bacterial load in sputum (WHO, 2009). Finally, in patients suspected of extrapulmonary TB, an antigen detection test might prevent the use of more invasive tests (WHO, 2009; Flores et al., 2011). These characteristics, if paired with an appropriate and simple method for antigen detection, make this approach applicable at the community level of the health system, so major efforts are being made to identify pathogen-derived antigens excreted in urine (Choudhry and Saxena, 2002).

One of the most promising antigens that are being evaluated is lipoarabinomannan (LAM). LAM is a structurally important component of the outer cell wall of all bacteria of the genus *Mycobacterium* that is shed from metabolically active or degrading cells, is cleared by the kidney and detectable in urine (Hunter et al., 1986; Chan et al., 1991). Antigen detection assays were described for LAM, most of which are based on a sandwich capture ELISA format to detect LAM in sputum (Pereira Arias-Bouda et al., 2000) or urine (Hamasur et al., 2001; Boehme et al., 2005; Mutetwa et al., 2009). As it will be described below, this antigen is being currently evaluated by a lateral flow test for rapid LAM detection in urine (Minion et al., 2011).

In addition to LAM, defined *M. tuberculosis* protein antigens were assayed as target for antigen detection assays. The tested proteins are generally major components that have been identified by electrophoresis and mass spectrometry both in total extracts and culture filtrate of *M. tuberculosis* (Malen et al., 2007; Mattow et al., 2003). **Table 1** summarizes available information from these antigens provided in TB Genomes Database (Reddy et al., 2009) and TubercuList (Lew et al., 2011). It is important to mention that many former published studies described the use of an antigen based only upon its apparent molecular weight on SDS-PAGE and without further identification of the protein. This is the case of a 55 kDa antigen (Rv not reported) present in serum of pulmonary (Attallah et al., 2003) and extra-pulmonary TB patients (Attallah et al., 2005), and a 20 kDa antigen (Rv not reported) detected in MTB crude extracts and serum of pulmonary TB patients (El-Masry et al., 2008).

**Table 1:** Summary of principal protein antigens evaluated for *Mycobacterium tuberculosis* direct diagnosis.

Gene	Rv number	Protein information (alternative nomenclature)	Function	Diagnostic evidence
*apa*	Rv1860	Alanine proline rich secreted protein APA (immunogenic protein MPT32; 45-kDa glycoprotein; 45/47 kDa antigen).	Unknown (could mediate bacterial attachment to host cells).	Tested in sputum and serum of active smear-positive TB patients (Chanteau et al., 2000).
*esxA*	Rv3875	6 kDa Early secretory antigen target ESXA (ESAT-6).	Elicits high level of IFN-gamma from memory effector cells during first phase of a protective immune response. Co-transcribed with Rv3874 (CPF10).	Detected in cerebrospinal fluid (CSF) of tuberculous meningitis patients (Kashyap et al., 2009).
*fbpA*	Rv3804c	Secreted antigen 85-A FBPA (mycolyl transferase 85A; fibronectin-binding protein A; antigen 85 complex A)	Involved in cell wall mycoloylation. Proteins of the antigen 85 complex are responsible for the high affinity of mycobacteria to fibronectin. Possess a mycolyltransferase activity required for the biogenesis of trehalose dimycolate (cord factor), a structure necessary for maintaining cell wall integrity.	Antigen 85 complex proteins have been detected in sputum (Wallis et al., 1998) and serum (Kashyap et al., 2007) specimens of TB patients.


*fbpB*	Rv1886c	Secreted antigen 85-B FBPB (mycolyl transferase 85B; fibronectin-binding protein B; antigen 85 complex B)		
*fbpD*	Rv3803c	Secreted MPT51/MPB51 antigen protein FBPD (MPT51/MPB51 antigen 85 complex C; mycolyl transferase 85C; fibronectin-binding protein C)		
*glcB*	Rv1837c	Malate synthase G (GlcB)	Involved in glyoxylate bypass, an alternative to the tricarboxylic acid cycle.	Assayed with promising results in CSF in tuberculous meningitis (Haldar et al., 2012).
*groEL2*	Rv0440	60 kDa chaperonin 2 GROEL2 (GROEL protein 2; 65 kDa antigen; heat shock protein 65)	Prevents misfolding and promotes folding and proper assembly of unfolded polypeptides.	Showed a good diagnostic performance in ELISA of serum samples of TB patients (Rajan et al., 2007)
*hspX*	Rv2031c	Heat shock protein HSPX (alpha-crystallin homolog; 14 kDa antigen; 16 kDa antigen; HSP16.3)	Stress protein induced by anoxia. HSPX has a proposed role in maintenance of long-term viability during latent, asymptomatic infections, as well as in replication during initial infection.	Assayed with promising results in CSF in tuberculous meningitis (Haldar et al., 2012)
*moeX*	Rv1681	Possible molybdopterin biosynthesis protein MoeX	Involved in molybdopterin cofactor biosynthesis.	Identified by mass spectrometry in urine from active tuberculosis patients (Pollock et al., 2013)
*mpt64*	Rv1980c	24 kDa immunogenic protein MPT64 (antigen MPT64/MPB64).	Secreted protein of unknown function specific for *M. tuberculosis* complex. Highly secreted during initial phases of bacterial growth.	A lateral flow assay was developed for the identification of *M. tuberculosis* complex in liquid culture media by using anti-MPB64 monoclonal antibodies (Akyar et al., 2010)
*pstS1*	Rv0934	Periplasmic phosphate-binding lipoprotein PSTS1 (PBP-1; immunodominant 38 kDa protein; protein antigen B).	Involved in active transport of inorganic phosphate across the membrane (Chang et al., 1994).	Assayed in CSF in tuberculous meningitis (Haldar et al., 2012)
*TB31.7*	Rv2623	Universal stress protein family protein TB31.7.	Regulates mycobacterial growth and is required for the entry of tubercle bacillus into the chronic phase of infection (Drumm et al., 2009).	Potential biomarker for the diagnosis of latent as well as active tuberculous meningitis infection. Assayed in CSF (Jain et al., 2013).

*Table includes data derived from *TB Genomes Databases* (Reddy et al., 2009) and *TubercuList* (Lew et al., 2011). Antigens are alphabetically ordered by gene name.*

More recently, high throughput approaches were designed to facilitate new biomarker discovery, based on proteomic approaches employing clinical samples from active TB patients. Thereby, four *M. tuberculosis* proteins were detected in urine samples, which were identified as a possible molybdopterin biosynthesis protein MoeX (Rv1681), a probable ornithine carbamoyltransferase ArgF (Rv1656), a probable homoserine O-acetyltransferase MetA (Rv3341) and a probable 3′-phosphoadenosine 5′-phosphosulfate reductase CysH (Rv2392; Kashino et al., 2008; Napolitano et al., 2008). These proteins constitute interesting candidates for the development of antigen detection assays, and recently the gene coding for MoeX, unique to the *M. tuberculosis* complex, was clinically validated as a diagnostic biomarker for active pulmonary TB (Pollock et al., 2013).

## DIAGNOSTIC PERFORMANCE AND COMMERCIAL DEVELOPMENT

A small number of commercial prototype TB diagnostic tests based on antigen detection have been developed, and some of them were evaluated for clinical diagnostic performance. These tests includes: Patho-TB (Anda Biologicals, France), Diagnos TB Ag (Biomed Industries, India), LAM-ELISA (Chemogen, USA, a prototype test not currently available), Clearview TB ELISA (Inverness Medical Innovations, USA) and Determine TB-LAM (Alere Inc., USA; Flores et al., 2011; Minion et al., 2011). Two recent systematic reviews and meta-analysis highlighted that these tests showed heterogeneous values of sensitivity and specificity through clinical evaluations (Flores et al., 2011; Minion et al., 2011).

Some of these tests were designed to detect different *M. tuberculosis* antigens in sputum. Patho-TB rapid diagnostic test uses polyclonal antibodies to detect mycobacterial antigens (including 65 and 85 kDa antigens) in sputum samples, previously decontaminated and imprinted in a filter cartridge. This test showed a sensitivity ranging from 90 to 96% and specificity between 70 and 100% in different evaluation studies (Fabre et al., 2007; Alavi-Naini et al., 2009; Ben-Selma et al., 2009). Another rapid test designed to detect mycobacterial antigens in sputum [including LAM and antigen 85B (Rv1886)] using polyclonal antibodies is Diagnos TB Ag. The test comprises the inactivation and lysis of the sputum sample, loading of the sample on a membrane device and immune-detection of specific antigens. It showed a variable performance in two published studies: a sensitivity of 98% and specificity of 99% in TB infected HIV sero-positive patients (Chakraborty et al., 2009) versus a 60% sensibility and 33% specificity in HIV positive and TB negative infected patients (Reither et al., 2010).

Another group of tests is based on LAM detection in urine. Diagnostic tests based on the detection of LAM in urine were among the first to move from research to commercial stage, due to their promising initial results (Hamasur et al., 2001; Boehme et al., 2005). However, they have not yet been routinely applied in remote points of care settings (Minion et al., 2011; Pai and Pai, 2012). The conflicting results obtained with these tests can be explained, in part, by the lack of specificity of the anti-LAM antibodies, since even anti-LAM monoclonal antibodies cross-react with most species of *Mycobacterium*, including *M. avium* and *M. leprae* (Hunter et al., 1986; WHO, 2009).

The LAM-ELISA (Chemogen Inc., Portland, USA) was the first LAM targeting assayed prototype (Boehme et al., 2005; Daley et al., 2009; Lawn et al., 2009; Mutetwa et al., 2009; Reither et al., 2009). Afterward another commercial version named Clearview TB ELISA (Alere Inc., USA – formerly Inverness Medical Innovations, Inc.) was launched (Dheda et al., 2010; Shah et al., 2010). Both tests use polyclonal anti-LAM antibodies in a capture sandwich ELISA format. A meta-analysis of published clinical studies with different versions of these tests showed that 57% of urine samples from smear positive TB patients were positive for LAM, indicating that this test would not be sufficiently sensitive to replace sputum microscopy. Nevertheless, 41% of smear negative TB patients were positive for LAM, suggesting that LAM testing and sputum microscopy used together could help diagnose different groups of patients with TB (Minion et al., 2011). In addition, it has been reported that a pre-analytical 100-fold-concentrating step of urine samples increased significantly the sensitivity of the Clearview TB ELISA (Savolainen et al., 2013), yet the method still needs to be refined to become a viable tool for TB diagnosis.

Promisingly, a POC lateral flow dipstick version of urinary LAM detection (Determine TB LAM Ag, Alere Inc.) has been developed. Following its commercial launch in 2013, Determine TB-LAM remains the focus of ongoing clinical evaluation studies. This is a simple, low-cost, POC assay which provides a qualitative (yes/no) readout of TB diagnosis within 30 min (Lawn, 2014). While diagnostic evaluation of this kit showed a poor performance with unselected TB patients, combination of LAM lateral flow test with sputum microscopy demonstrated a diagnostic value in HIV immunocompromised TB patients (CD4 lymphocyte cell counts <50/μL; Lawn et al., 2012; Peter et al., 2012). These and other studies largely confirm that the sensitivity of Determine TB-LAM is greatest (range 60–70%) among HIV-infected patients with the most advanced immunodeficiency (Lawn et al., 2013). HIV-associated pulmonary TB is of major concern in many countries of Africa, thus this kit could assist to establish a quicker diagnosis and an earlier treatment in this high risk population. As the evidence base grows, data on this assay would be reviewed by an expert panel convened by WHO to define the role of the assay as an add-on test within existing diagnostic algorithms (Lawn et al., 2013).

## CONCLUSION

A reliable POC diagnostic test for active TB detection is urgently needed and much work is being done for that purpose. In the TB diagnostic field, antigen detection technologies and biomarker discovery strategies are rapidly evolving. While there are promising evidences endorsing some of the commercially available diagnostic tests, none of the new tools designed so far have shown an outstanding diagnostic performance as to promote its widespread application in medical practice. It is highly expected that in the coming years more light is shed to aid in that goal.

## Conflict of Interest Statement

The authors declare that the research was conducted in the absence of any commercial or financial relationships that could be construed as a potential conflict of interest.
